# Domestication process modifies digestion ability in larvae of Eurasian perch (*Perca fluviatilis*), a freshwater Teleostei

**DOI:** 10.1038/s41598-020-59145-6

**Published:** 2020-02-10

**Authors:** Katarzyna Palińska-Żarska, Maciej Woźny, Maciej Kamaszewski, Hubert Szudrowicz, Paweł Brzuzan, Daniel Żarski

**Affiliations:** 1 Department of Ichthyology and Aquaculture, University of Warmia and Mazury, Oczapowskiego 5, 10-719 Olsztyn, Poland; 2 Department of Environmental Biotechnology, University of Warmia and Mazury, Słoneczna 45G, 10-709 Olsztyn, Poland; 3 Departament of Ichthyology and Biotechnology in Aquaculture, Institute of Animal Sciences, University of Life Sciences, Ciszewskiego 8, 02-786 Warsaw, Poland; 4 Department of Gametes and Embryo Biology, Institute of Animal Reproduction and Food Research, Polish Academy of Sciences, Tuwima 10, 10-748 Olsztyn, Poland

**Keywords:** Animal physiology, Ichthyology

## Abstract

To date, a comparative analysis of larval performance and digestion abilities between wild and domesticated Eurasian perch has not yet been performed. Eurasian perch larvae from wild and domesticated spawners were reared in the same conditions and at different development stages, growth performance variables, the expression of genes encoding digestive enzymes and specific enzymatic activity were analysed. No significant differences in hatching rate, deformity rate or swim bladder inflation effectiveness between wild and domesticated larvae were found. Specific growth rate, final total length and wet body weight were significantly lower in wild larvae, whereas higher mortality in wild larvae was observed compared to domesticated larvae. The data obtained in this study clearly indicate that during domestication, significant modification of digestion ability occurs at the very beginning of ontogeny, where domesticated fish are characterised by lower enzymatic activity and lower expression of genes encoding digestive enzymes. This probably results from the low diversity of the food offered in culture conditions, which significantly modified digestion capability. The obtained data provide an understanding of how domestication affects fish in aquaculture and may improve the planning of selective breeding programs of Eurasian perch and other freshwater Teleosts.

## Introduction

Domestication of animals involves transformation of morphological, physiological, developmental and behavioural features, during which animals adapt to the environment that humans create for them^1^. Domestication was indicated as a key element allowing sustainable expansion of aquacultural production^2^, being the priority of the sector in the view of ever-growing demand of the consumers, overexploitation of nearly 80% of global fish stocks and a constantly increasing human population^3,4^. However, despite the huge importance of domestication for the aquaculture industry, there is very little known of the processes being modified along with the adaptation of the fish to the culture conditions. This applies especially to the most intensive farming technologies, such as those involving a recirculating aquaculture system (RAS), where most of the environmental factors, to which fish are exposed throughout the life cycle, are fully controlled by humans^5^.

One of the most important species dedicated to RAS-based aquaculture production is the Eurasian perch *Perca fluviatilis* L., whose domestication began in the 1990s^6^. It is accepted that some cultured stocks of this species are at the 4^th^ level of domestication – the entire life cycle is closed in intensive culture conditions, without inputs of wild specimens, but there are still no selecting breeding programs successfully implemented^2,6^. The Eurasian perch is an ideal model for research on domestication processes because this is the only native European species where several generations with a clearly indicated history of origin are farmed in RAS^7,8^ and, at the same time, there is still easy and wide access to the wild fish from many different populations^9^. Essentially, effective production of this species relies solely on domesticated, RAS-grown stocks characterized by variable spawning and growth performance in the culture conditions. This can stem from: (i) different populations being domesticated or (ii) different practices leading to exhibition of different traits in captivity. It is already known that different populations can show different growth^10,11^ and behavioural traits^12^, however, there is no information on which processes are conditioning production effectiveness. Therefore, knowledge of the processes conditioning adaptability of this species to the culture conditions, along with their domestication, are crucial elements toward the development of future breeding programs.

The larval period of finfishes, despite being the shortest period in ontogeny, along with embryonic development, is also crucial for every species. The functional and morphological aspects of larvae development show that larvae are very different organisms during this period when compared to adult fish^13^. In addition, the most significant changes in fish morphology, physiology and behaviour occur during this period^14,15^, e.g. rapid changes in body weight and length^16^, starting of exogenous feeding, oil droplet reduction^17^ and filling of the swim bladder^18,19^. All of these are key events that ensure the success of intensive larviculture of most fish species and determine the effectiveness of commercial production. Moreover, considering domestication as a specific process during which the exhibition of particular traits occurs – conditioning adaptability of the fish to the culture conditions – this process starts precisely during the larval period. However, to date, there has been little information on the processes conditioning larval survival and growth performance in finfish stemming from the domestication level. It was previously reported that there are significant differences in the transcriptomic profile of eggs^20^ and embryos^21^ from domesticated and wild fish, where most genes involved in immune and stress response were significantly modified by the domestication. However, comparison of transcriptomic profile of wild and domesticated adult Eurasian perch suggested that the domestication process may also significantly modify the digestion capacity^22^. However, due to the lack of evidence at the embryonic and/or larval phase on the domestication-related modifications of the digestion-related processes, there is a high need to investigate whether the differences in digestion capacity are exhibited in the very early phases of larval development or if the differences stem from long-term exposure to a different diet. In other words, there is a lack of clear evidence whether the digestive capacity can be ‘programmed’ along with domestication, during which fish are exposed to alternatives to naturally available types of food. Such ‘programming’ was thought to be important feature conditioning larval adaptation^23^. It should be emphasized that there is a lack of published information on the effect of the domestication process on larval performance and digestion capacity in finfish, including the Eurasian perch.

In most cases, it is known that domestication of animals leads to the exhibition of biological features desired by humans^24,25^ and that nutrition is one of the main factors conditioning adaptive and evolutionary abilities^26^. Until today, in fish, it is known that domesticated fish grow faster^27^ and that their behaviour changes^12^, they were more resistant to stress^28–30^ and domestication could cause changes in fish digestive systems^28^ and their gene expression^31^. Knowledge of the mechanisms conditioning the process of domestication and the impact of domestication on the rearing of fish is still very limited^32–34^ as is the knowledge available on whether/how domestication affects fish larvae^35,36^. In addition, biological variables of the development of larvae from wild and domesticated specimens have not yet been compared. It is also still unclear which features result from domestication and which result simply from the phenotypic plasticity of a particular fish species. Therefore, there is an urgent need for the collection of data on all of these aspects, which would make a significant contribution to knowledge of the domestication process in fish.

The ontogeny of the digestive system of finfish is already well described^37^, as well as the digestive system of percid fish^38^. It is well known that the fish larvae, including percids, are able to secrete some digestive enzymes just after hatching^23,39–41^. However, during the first days of exogenous feeding, the digestion is characterized by low efficiency and needs to be supported by exogenous enzymes derived from the ingested prey^13^. That is why in intensive larviculture the larvae need to be fed first with live *Artemia* sp. nauplii, being the source of both – indispensable nutrients and exogenous enzymes^13,42^. Along with the resorption of the yolk-sac, the production of digestive enzymes increases^41,42^ and – to a certain extent – their composition and amount adapt to the type of food available^43^. Despite the fact that temperature has a modulatory effect on the production of digestive enzymes, the main factor conditioning the qualitative and quantitative composition of the digestive enzymes is the type of the food^43,44^ and genetic factors^23^, with the latter being sparsely studied in finfish^45^. It should be noted that along with the domestication process, a specific gene pool is being preserved and there is an urgent need to investigate its influence on the digestive capacity of fish larvae.

Within the study, a comparative analysis of larval performance between wild and domesticated Eurasian perch was performed. A further comparison included an evaluation of digestion abilities, which was thought to be linked with the adaptability of fish larvae to intensive culture conditions.

## Materials and Methods

### Obtaining perch larvae

Wild Eurasian perch larvae were obtained by controlled reproduction of wild spawners from the ‘Żurawia’ Fish Restocking Centre (Central Poland). For reproduction, 8 females (average weight 430 ± 30 g) and 24 males (average weight 124 ± 60 g) were used. Spawners were collected from earth ponds in October and after four months of wintering in a flow-through pond system (including a minimum of two months at a temperature below 6 °C) delivered to the laboratories of the Centre of Aquaculture and Ecological Engineering of the University of Warmia and Mazury (NE, Poland). Shortly after transport, fish were placed in tanks equipped with devices for temperature (±0.1 °C) and photoperiod (±10 min) control and were acclimated at 10 °C for a 5-day period. After this time, the temperature in the tanks was raised to 12 °C over two days. Hormonal stimulation was then used (a single human chorionic gonadotropin [hCG] injection at a dose of 500 IU kg^−1^). After injection, fish were kept at 12 °C until ovulation. Eggs (ribbons) were obtained at ovulation (according to the method described by Żarski *et al*.^46^) and fertilised *in vitro* (15 s and 30 s after activation with water) as described by Żarski *et al*.^47^. Eggs from one female were fertilised using sperm obtained from four males. Eggs from each female were incubated separately.

Eggs (ribbons coming from eight females) of domesticated Eurasian perch were obtained from fish from the F5 generation (the 5^th^ generation reared in controlled conditions) from a commercial fish farm from France (Lorraine) which reared perch in RAS. Eggs from domesticated females were obtained with the same procedure as those from wild fish (hormonally injected with 500 IU of hCG kg^−1^ and were stripped and fertilised *in vitro* with sperm coming from at least 4 males). Next, 36 h after fertilisation, eggs were transported to the laboratories of the Centre of Aquaculture and Environmental Engineering in bags with water and oxygen and then placed in the experimental RAS.

The same incubation procedures were used for eggs obtained from wild and domesticated spawners. Eurasian perch ribbons were incubated (ribbons from each female were always incubated separately) on submerged nets (with mesh diameter around 1 mm)^48^ at 14 °C, in 20 L black tanks working within the same RAS. Experimental RAS was provided with biological filtration, automatically regulated photoperiod (±10 min) and temperature (±0.1 °C). Ribbons from each female were incubated in two separate tanks (a total of 16 tanks for ribbons originating from eight females). When black pigmentation in the eyes of perch embryos was observed (the eyed-stage), the water temperature was raised to 15 °C and when the first hatching larvae were observed in the tanks, the temperature was raised again to 16 °C. After spontaneous hatching (which lasted about 12 h), the nets with unhatched eggs were removed from the tanks. This moment was considered the end of hatching (0 Days Post Hatching - DPH). To ensure the maximum homogeneity of perch larvae in terms of size, stage of development and their quality, no manual hatching (extraction) was induced. After hatching was completed, larvae were taken out of tanks into a calibrated container and tanks were cleaned to remove any leftovers. Next, larvae were counted volumetrically and stocked back (larvae from each female separately, in triplicates) to the tanks, at a density of 10,000 ind. per tank.

### Rearing of wild and domesticated larvae and variables tested

The same rearing procedures were applied for larvae obtained from wild and domesticated Eurasian perch. Larvae were reared in the same tanks in which the eggs were incubated. One day following hatching (1 DPH) water temperature in RAS was raised to 17 °C and on 2 DPH once more to 18 °C. Two days after oil droplet reduction in at least 50% of the larvae, the temperature in the RAS was raised gradually (1 °C every day) to 23 °C (considered the optimal temperature)^49^.

Throughout the entire rearing period, a photoperiod of 24 D:0 L was used, and the intensity of light, measured at the water surface was 1500 lux. The concentration of ammonia was tested every two days (Hach DR800, USA) and it never exceeded 0.01 mg L^−1^. The oxygen concentration was also tested twice per day (with a Handy Polaris 2.0 OxyGuard, Denmark, oxygen probe) and a decrease below 80% oxygen saturation was never recorded. All tanks were provided with water from the top-inlet gently spraying the water surface, but no ‘surface skimmer’ was applied.

**Figure 1:**
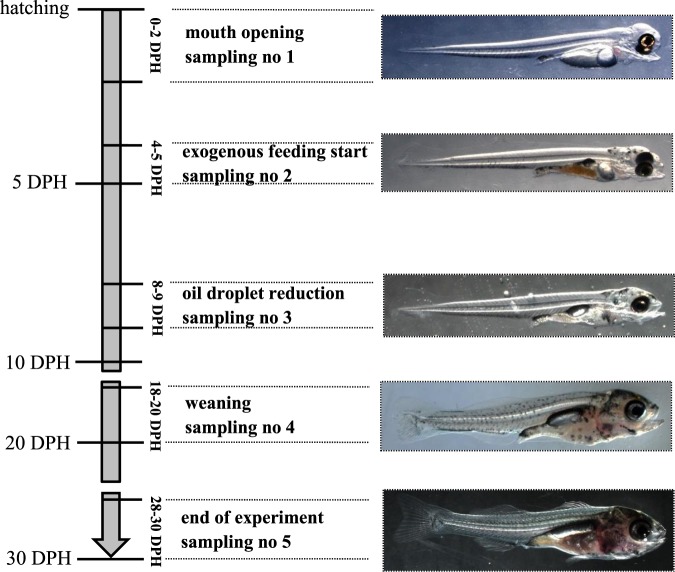
Scheme of Eurasian perch larvae samplings (same for wild and domesticated ones) at selected development steps. DPH – Days Post Hatching, sampling no 1 – moment of mouth opening in at least 50% of perch larvae, sampling no 2 – moment of exogenous feeding starting in at least 50% of perch larvae, sampling no 3 – moment of oil droplet reduction in at least 50% of perch larvae, sampling no 4 – moment of weaning onto dry diet, sampling no 5 – end of experiment (10 days on dry diet).

At certain development stages of the larvae, samples were taken to determine the biological variables, digestive enzyme mRNA expression and specific enzymatic activity (Fig. 1):

1. Mouth opening in at least 50% of the larvae (0–2 DPH),
2. When exogenous feeding starts in at least 50% of the larvae (4–5 DPH),
3. Oil droplet reduction in at least 50% of the larvae (8–9 DPH),
4. Weaning (18–20 DPH),
5. The end of the experiment − 10 days on dry feed (28–30 DPH).

The total length (TL, ±0.01 mm) of Eurasian perch larvae was measured with the use of a stereoscopic microscope (Leica, Germany) and wet body weight (WBW, ±0.1 mg) was determined using a precision laboratory scale (Ohaus, USA). Two days after oil droplet reduction, the swim bladder inflation efficiency (SBIE%) was calculated. The SBIE was determined using a stereoscopic microscope, by triple counting - on a Petri dish - perch larvae (with and without a filled swim bladder) randomly caught from each tank (in total, SBIE was determined from more than 100 larvae from each tank). In addition, WBW measurements were performed by the so-called ‘non-invasive method’^50^. For this purpose, anaesthetised larvae were placed on a platform made of a nylon mesh (with a mesh size of approx. 200 μm) and excess water was drained by filter paper. This method minimises possible physical damage to very delicate larvae. Before all manipulations, the larvae were anaesthetised in a solution of MS-222 (at a dose of 150 mg L^−1^).

At hatching, the percentage of hatched larvae (%) and the percentage of deformation (%) were determined. Based on the WBW measurements, the specific growth rate (SGR, % d^−1^) was also calculated^51^:

$${\rm{SGR}}=100\times (\mathrm{ln}\,{\rm{W2}}\,-\,\mathrm{ln}\,{\rm{W1}}){\Delta {\rm{t}}}^{-1},$$

where: W2 - average body weight at the end of the experiment (mg), W1 - average body weight at the time of hatching (mg) and Δt - time of rearing (days).

Starting from 2 DPH, about 200 larvae were placed every day into a glass beaker (1 L) and fed with *Artemia* sp. (Ocean Nutrition, Belgium). When more than 50% of larvae in the beaker started exogenous feeding, the rest of the perch larvae began to be fed (4–5 DPH). The larvae were fed with *Artemia* sp. nauplii three times a day, *ad libitum* (first four days of feeding – micro *Artemia* cysts, than standard size *Artemia* cysts, 260000 nauplii per gram). After weaning, Eurasian perch larvae were fed with dry feed (Perla Larva Proactive, Skretting, Norway) six times a day, pouring it into each tank in small doses for about 15 minutes. After each feeding, the tanks were cleaned and dead individuals were counted.

### Extraction and purification of total RNA, and synthesis of cDNA

Immediately after collection, the larvae were preserved in an RNAlater solution according to the manufacturer’s recommendations (Sigma-Aldrich, Germany). Total RNA was extracted from approximately 20 mg of the preserved tissue using a Total RNA mini-kit (A&A Biotechnology, Poland). The extracted samples were incubated with TURBO DNAse (Invitrogen, USA) and then purified using a Total RNA mini-kit (A&A Biotechnology). To eliminate genomic DNA, the RNA quantity and purity were measured with a NanoDrop 8000 spectrophotometer (Thermo Fisher Scientific, USA). Samples with absorbance ratios A_260/280_ < 2.0 and A_260/230_ < 2.2 were further selected for reverse transcription. To check the integrity of the extracted total RNA, a representative group of samples (n = 12) were additionally examined with an Agilent Bioanalyser 2100 (Agilent Technologies, USA) and found to have RIN values >7.

Reverse transcription was carried out using a RevertAid First Strand cDNA synthesis kit (Thermo Fisher Scientific). The cDNA synthesis reaction contained 2 μg of the purified DNAse-treated total RNA and 5 μM of oligo(dT)_18_ primer. After optional incubation (65 °C for 5 min), the samples were chilled on ice and the following components were added: 4 µL of 5X Reaction Buffer, 20 U of RiboLock RNAse Inhibitor, 1 mM of dNTP mix, and 200 U of RevertAid M-MuLV Reverse Transcriptase. To check for DNA contamination of RNA samples, an additional control reaction was run with all of the components except the enzyme (RT−). The reaction was carried out at 42 °C for 60 min and then terminated by heating at 70 °C for 5 min. Synthesised cDNA samples were diluted (1:2) and stored at −80 °C and thawed only once, just before amplification.

### PCR primers design

Primer pairs for all mRNA targets (except pepsinogen: *pga*) for the RT-qPCR study were designed using a Primer-BLAST software^52^ based on Eurasian perch mRNA sequences downloaded from PhyloFish database (http://phylofish.sigenae.org)^53^. Because it was not possible to find a percid *pga* mRNA in this database, a PCR primer pair was designed based on the *pga* mRNA sequence of the gilt-head bream (*Sparus aurata*) found in Genbank (accession number EU163284) and used in conventional PCR with Eurasian perch cDNA to amplify perch-specific fragments using DreamTaq Green PCR Master Mix (Thermo Fisher Scientific). Homogenous amplicons of 484 bp were cloned using a TA Cloning Kit (Invitrogen) and were sequenced under contract (Genomed, Poland). The obtained sequence indicated an open reading frame of 138 amino acids, which showed high similarity to pepsinogen of different animal species. The obtained perch *pga* mRNA sequence was deposited in GenBank (under accession number MH509190) and further served as templates to design the final set of RT-qPCR primers for this transcript (Table 1).

**Table 1:** Details of the RT-qPCR primers used in the study.

Gene name	Sequence [5′ → 3′]	Amplicon length [bp]	Optimal concentration of each primer [nM]	Reference
Adenosine kinase-like [*adk*]	F: CTTCCTGACCGTCTCTTTGG	209	500	http://phylofish.sigenae.org/ngspipelines/report.jsp?id=21732&root=NGSpipelines&datasetType=rnaseqDenovo&plugin=contigsreport&mart=Perca%20fluviatilis
	R: CCTTGGTCTCGAAGTCTTGC			
Alpha-amylase [*amy*]	F: CCGGTGACCTGTCTGCTATC	112	250	http://phylofish.sigenae.org/ngspipelines/report.jsp?id=38665&root=NGSpipelines&datasetType=rnaseqDenovo&plugin=contigsreport&mart=Perca%20fluviatilis
	R: GGCTCACCTCCCAAGTCAAT			
	R: TCGTCCTCGGCTTTGTATCG			
Lipoprotein lipase [*lpl*]	F: CAAACTGGTGTCGGCTCTCTAT	120	250	http://phylofish.sigenae.org/ngspipelines/report.jsp?id=22657&root=NGSpipelines&datasetType=rnaseqDenovo&plugin=contigsreport&mart=Perca%20fluviatilis
	R: CGGCCGACTAGTTTAGTGTCA			
	R: TCAAAGTCCACTGTGAGCGG			
Pepsinogen [*pga*]	F: CCACCTACTGGCAGATCTCA	174	500	https://www.ncbi.nlm.nih.gov/nuccore/MH509190
	R: CATCTCCGTACTGGTCGGTT			
Trypsin [*try*]	F: GACACCGGGGTTGTTCTTCT	137	500	http://phylofish.sigenae.org/ngspipelines/report.jsp?id=21025&root=NGSpipelines&datasetType=rnaseqDenovo&plugin=contigsreport&mart=Perca%20fluviatilis
	R: TGTTCTGCGCTGGATACCTG			

### Real-time PCR

RT-qPCR was performed on a Quant Studio 5 (Applied Biosystems, USA) in a singleplex mode. Each PCR reaction tube contained 5 μL of 2X Power SYBR Green Master Mix (Applied Biosystems), an optimised concentration of forward and reverse primer (Table 1), 0.5 µL of the diluted cDNA as a template, and PCR-grade water for a final volume of 10 μL. The reaction was performed in duplicate in standard thermal conditions: 95 °C for 10 min, then 40 cycles of 95 °C for 15 s and 60 °C for 1 min. On the plate, no template control (NTC) or RT-negative samples were included to rule out the possibility of cross-contamination. A melting curve analysis (followed by agarose gel electrophoresis) was performed to verify the quality of the obtained PCR products (Supplement 1). Finally, the specificity of the products was confirmed by cloning (TA Cloning Kit, Invitrogen) and sequencing representative amplicons (Genomed, Warsaw, Poland).

In order to assess RT-qPCR efficiencies, plasmids with target sequences were used to prepare a series of six 10-fold dilutions and served as the template in qPCR. Cq values obtained for each dilution were plotted against the log of the plasmid DNA concentration and were then used to generate a linear function. Based on the results obtained for the functions of standard curves, PCR efficiency was calculated according to the following equation^54^:

$$E={10}^{(\frac{-1}{slope})}$$

Analysis of Cq values with BestKeeper^55^ indicated adenosine kinase-like (*adk*) and heat shock protein (*hsp70*) to have the lowest variation among all RT-qPCR primer pairs. In turn, additional analysis of the data with geNorm software^56^ identified *adk* and lipoprotein lipase (*lpl*) as the most stable primer pairs. Since the *adk* showed acceptable expression variance across all of the analysed cDNA samples in both evaluations (Supplement 1), it was used to normalise the expression of mRNA targets, according to the following equation derived from Livak and Schmittgen^57^:

$$normalized\,expression=\frac{1}{{E}^{(C{q}_{target}-C{q}_{reference})}}$$

### Enzyme activity analysis

Just after collection, perch larvae were frozen in liquid nitrogen and then stored at −80 °C. At mouth opening, when exogenous feeding starts, oil droplet reduction was observed and at weaning the whole larvae were frozen but, at the end of the experiment, the larvae were deprived of their heads and tail part, just before they were put in liquid nitrogen. The digestive enzyme activities assay of alpha-amylase (*amy*; EC 3.2.1.1), lipoprotein lipase (*lpl*; EC 3.1.1.3), trypsin (*try*; EC 3.4.21.4), leucine aminopeptidase (LAP; EC 3.4.11.1) and pepsin (EC 3.4.23.1), to evaluate protein levels samples of perch larvae were first homogenised in buffers and then centrifuged at 4 °C for 15 min. at 15000 × g. The specific enzyme activity was analysed according to the procedures described for amylase^58^, lipase^59^, trypsin^60^, LAP^61^ and pepsin^62^. Protein content was evaluated according to Lowry *et al*.^63^. Analyses of enzyme activities for amylase, lipase, trypsin, LAP and pepsin were performed in five replicates at 25 °C. Enzyme activities were expressed as the number of micromoles of the reaction product per 1 minute calculated for 1 mg of protein (U/mg protein). Absorbance was measured using an Infinite 200 Pro, Tecan (Tecan Austria, Grödig, Austria).

### Data analysis and statistics

The statistical differences were analysed with a two-way analysis of variance (ANOVA) and Tukey’s *post hoc* test at a significance level below 5% (p < 0.05). Before analysis, data expressed in the percentage values were *arcsine* transformed. The statistical analysis was performed with Microsoft Excel and STATISTICA (data analysis software system) version 10 (StatSoft Inc., USA).

### Ethics approval and consent to participate

Authors confirm that all procedures and activities performed during experiment were carried out in accordance with Polish law (Act of January 15, 2015 on the protection of animals used for scientific or educational purposes) and following official decision of the Local Ethical Committee for Animal Experiments (Olsztyn, Poland) (LKE.065.12.2017) was approved.

## Results

### Biological variables

There were no significant differences in hatching rate, deformity rate and SBIE - between wild and domesticated Eurasian perch larvae (p < 0.05). However, the specific growth rate (SGR) was significantly (p < 0.05) lower in wild larvae − 9.12 ± 0.61% d^−1^, than in domesticated larvae 14.49 ± 0.63% d^−1^ and the mortality of wild larvae (24.8 ± 11.2%) was significantly higher (p < 0.05) than in domesticated larvae (10.2 ± 2.6%) (Table 2).

**Table 2:** Biological parameters obtained during rearing of Eurasian perch larvae. Data (mean ± SD) in columns marked with different letters were significantly different (p < 0.05). SBIE – Swim Bladder Inflation Effectiveness, SGR – Specific Growth Rate.

	Mortality (%)	SBIE (%)	Hatching rate (%)	Deformity rate (%)	SGR (% d^−1^)
Wild	24.78 ± 11.24^**a**^	41.29 ± 15.16^a^	62.5 ± 15.81^**a**^	5.4 ± 2.81^**a**^	9.22 ± 0.61^a^
Domesticated	10.22 ± 2.60^**b**^	33.75 ± 4.98^a^	63.75 ± 15.1^**a**^	4.0 ± 2.9^**a**^	12.80 ± 0.63^b^

**Figure 2:**
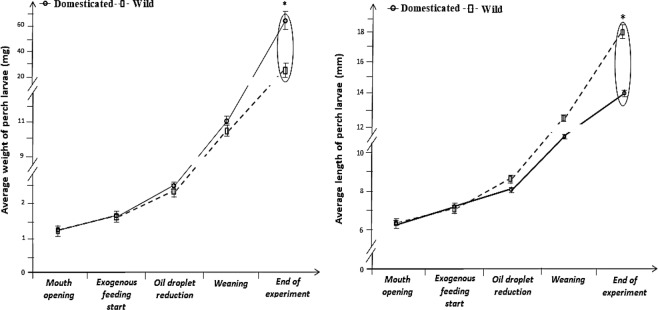
The average length (mm) and weight (mg) of Eurasian perch larvae at selected development steps (n = 90 domesticated and n = 90 wild larvae for determination of length and weight at each step were used). Data (mean ± SD), surrounded together and marked with an asterisk, were significantly different (p < 0.05).

Significant interactions were recorded between the developmental stage and the level of domestication in European perch larvae, in WBW and TL, following two-way analysis of variance (p < 0.05). At the moment of mouth opening, the WBW of wild larvae was 1.22 ± 0.09 mg and for the domesticated larvae it was 1.23 ± 0.05 mg (p > 0.05). There were no significant differences (p > 0.05) in the WBW of the larvae at the beginning of exogenous feeding, the moment of oil droplet reduction and at the moment of weaning. However, at the end of the experiment, the WBW of domesticated larvae were significantly higher (p < 0.05) than recorded in the wild larvae: 65.05 ± 13.73 mg and 24.05 ± 4.33 mg for domesticated and wild larvae, respectively (Fig. 2).

A similar trend as for WBW was observed for TL of Eurasian perch larvae, where at the moment of mouth opening, beginning of exogenous feeding, oil droplet reduction and at the moment of weaning, significant differences were not found (p > 0.05). However, at the end of the experiment, TL levels of domesticated larvae were significantly higher (18.28 ± 1.48 mm) than recorded in the wild larvae (14.09 ± 0.46 mm) (p < 0.05) (Fig. 2).

### Expression of genes encoding digestive enzymes

Lack of significant interactions between the developmental stage and the level of domestication in European perch larvae, in expression of genes encoding digestive enzymes, were recorded following two-way analysis of variance (p > 0.05).

#### *mRNA* level of genes encoding digestive enzymes in relation to developmental status of fish

**Figure 3:**
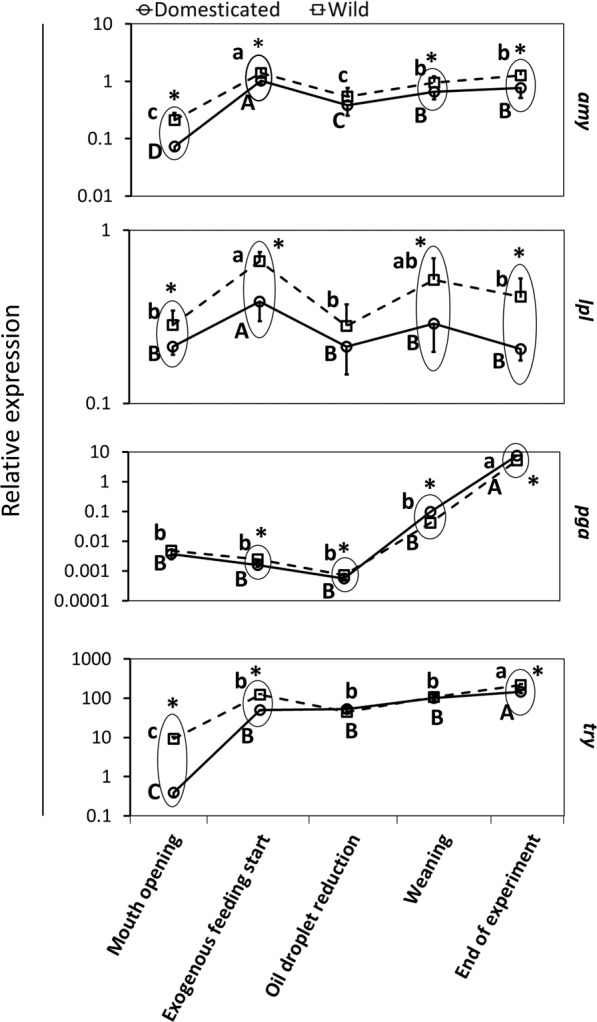
Dynamics of the digestive enzyme gene expression of Eurasian perch larvae at selected development steps. Data (mean ± SD) surrounded together and marked with an asterisk, were significantly different (p < 0.05) between wild and domesticated larvae. Different letters within the same group mean significantly differences (p < 0.05) – capital letters for wild Eurasian perch larvae and small letters for domesticated ones. *pga* – pepsinogen, *amy* – amylase, *lpl* – lipase, *try* – trypsin. Total RNA was extracted each time from approximately 20 mg of pooled tissue of perch larvae. Each group, at each sampling point, was represented by n = 8. Axis “Y” is presented as logarithmic (log_10_).

At the moment of mouth opening, significant differences in mRNA levels of *amy*, *lpl* and *try* were observed between wild and domesticated Eurasian perch larvae (p < 0.05). When exogenous feeding started, significant differences were observed in the mRNA level of all digestive enzymes (p < 0.05), while at the moment of oil droplet reduction this was observed only in pepsinogen (*pga*) (p < 0.05). At the moment of weaning, significant differences in wild and domesticated larvae were observed in *pga*, *amy* and *lpl* mRNA levels (p < 0.05), whereas at the end of the experiment significant differences were observed in the mRNA levels of all target genes (p < 0.05) (Fig. 3 and Supplement 2).

#### Dynamics of mRNA expression of genes encoding digestive enzymes

The expression of *amy*, *lpl*, *pga* and *try* was observed from the beginning of rearing in both wild and domesticated Eurasian perch larvae. At the moment exogenous feeding started, mRNA levels of *amy*, *lpl* and *try* sharply increased (p < 0.05), while *pga* mRNA levels did not differ (p > 0.05). At the moment of oil droplet reduction, the mRNA level of *amy* and *lpl* decreased (p < 0.05) while *pga* and *try* levels were unchanged compared to the moment of exogenous feeding starting (p > 0.05). After oil droplet reduction, the mRNA level of *amy* started to increase (p < 0.05) once more and the levels of the rest of the genes remained unchanged (p > 0.05) until the moment of weaning, in both wild and domesticated European perch larvae. At the end of the experiment, an increasing trend in mRNA expression of *amy* (in the case of domesticated larvae), *try* and *pga* was observed (p < 0.05), while *lpl* and *amy* (of wild larvae) mRNA expression remained unchanged (p > 0.05) (Fig. 3).

### Digestive enzyme activity

Two-way analysis of variance have revealed that there were no significant interactions in activity of digestive enzymes between the developmental stage and the level of domestication in European perch larvae (p > 0.05).

#### Specific activity of digestive enzymes in relation to developmental status of fish

**Figure 4:**
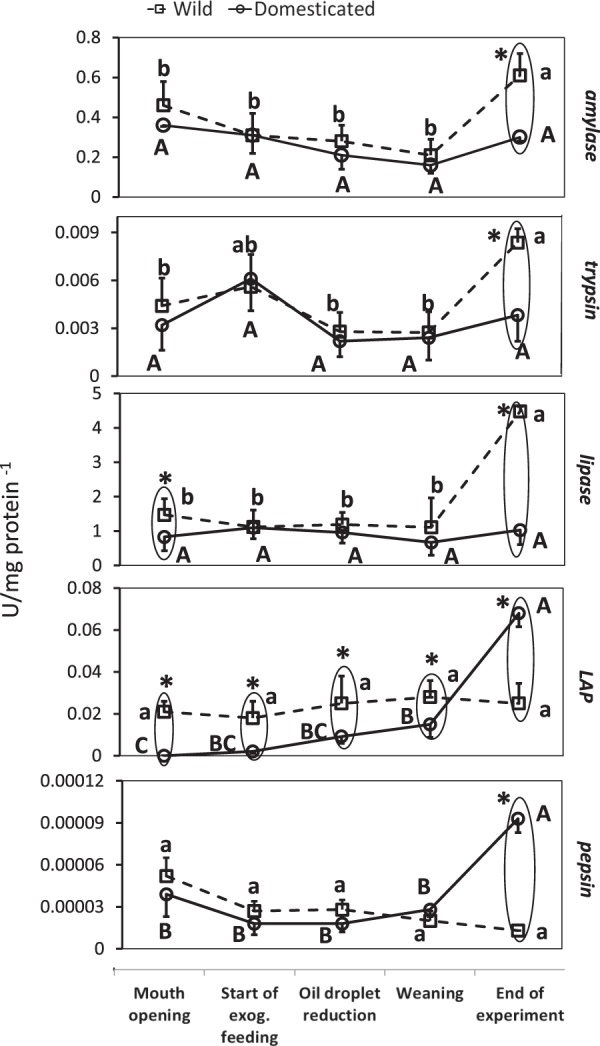
Dynamics of the digestive enzyme specific activity of Eurasian perch larvae at selected development steps. Data (mean ± SD) surrounded together and marked with an asterisk, were statistically different (p < 0.05) between wild and domesticated larvae. Different letters within the same group mean significant differences (p < 0.05) – capital letters for wild Eurasian perch larvae and small letters for domesticated ones. LAP – leucine aminopeptidase. To determine the specific enzymatic activity, 25 mg of tissue of perch larvae were used. Each group, at each sampling point, was represented by n = 8.

At mouth opening (when exogenous feeding starts), oil droplet reduction and, at weaning, significant differences (p < 0.05) were observed only in LAP specific activity between wild and domesticated Eurasian perch larvae. However, at the end of the experiment significant differences in the specific activities of amylase, trypsin, lipase and pepsin were observed (p < 0.05) (Fig. 4 and Supplement 3).

#### Dynamics of enzyme activity

Amylase, trypsin, lipase, pepsin (pepsin at a very low level, close to the detection limit of the assay) and LAP (only in wild Eurasian perch larvae) specific activities were detected as early as mouth opening. The specific activities of amylase and lipase showed a stable trend from the mouth opening to the moment of weaning (p > 0.05), both in domesticated and wild Eurasian perch larvae. At the end of the experiment, the specific activities of amylase and lipase (in wild larvae) increased sharply compared to their levels at mouth opening (p < 0.05), while the specific activity of lipase in domesticated Eurasian perch larvae remained unchanged (p > 0.05). The specific activity of trypsin in wild larvae reached its maximum at the end of the experiment and it was different from its specific activity at the moment of mouth opening (p < 0.05), while in domesticated Eurasian perch larvae the specific level of trypsin remained unchanged throughout the experiment (p > 0.05). The levels of specific activity of pepsin and LAP at mouth opening were almost undetectable (the specific activity of LAP in domesticated larvae was zero) and their specific activities remained at a very low level until weaning (p > 0.05). However, at the end of the experiment, the specific activities of pepsin and LAP increased sharply, but only in domesticated Eurasian perch larvae (p < 0.05), while in wild larvae they remained at an almost unchanged level (p > 0.05) (Fig. 4).

## Discussion

Rapid body growth is the first indicator of proper development and also the final result of several closely related processes such as, for example, efficiency of digestion and absorption of nutrients, metabolism and growth of all body cells, as well as neuroendocrine and endocrine control of these processes^27^. Many changes can take place in each of these processes and that is why differences in the growth of organisms are very often the most difficult to explain. Despite the fact that Eurasian perch aquaculture began to develop in the 1990s, there are still no standardised methods for rearing larvae in RAS (see^19^). That is why comparing obtained results with other authors is quite problematic although, in the current experiment, Eurasian perch larvae (both from wild and domesticated spawners) exhibited good larviculture performance and zootechnical rearing variables (for larvae obtained from wild spawners) and were comparable to those already reported by other authors, e.g. Tamazouzt *et al*.^64^, Baras *et al*.^49^, Kestemont *et al*.^49^ and Jentoft *et al*.^65^. This indicates that the rearing variables applied in the current study were optimal for this species.

It was reported that adult domesticated fish grow faster than wild fish of the same species^27,31^. However, there is no information on whether this trend also occurs in larvae^36^. The results of this study indicate that Eurasian perch larvae obtained from domesticated spawners exhibit better larviculture performance (final WBW and TL, SGR, SBIE, survival rate) when compared to those obtained from the wild spawners. However, it should be noted that the growth-related variables (WBW and TL gain) of the fish were comparable until the fish were offered an artificial diet. This suggests that larvae from wild and domesticated fish grew similarly until they were offered *Artemia* nauplii. Ingestion of compound feed requires more energy and involves more enzymes than the digestion of live food^13,66,67^. Although the larvae of wild fish grew slower following the changes in diet, it can be speculated that during the domestication process the main adaptation to the culture environment is the digestion capability of compound feed and that the offering of compound feed may be considered as a specific challenge for the larvae. Only the larvae which adapt to such a challenge can survive and later contribute to the new generation. This, in turn, indicates that domestication in fish (as in any other animal) is based on challenging the fish by the provision of unfavourable conditions. In the case of the larval period, the type of food may be considered as the main ‘challenging factor’. However, to confirm this hypothesis, more detailed research on the effect of differing qualities of compound feed on survival and growth performance between wild and domesticated fish larvae is required.

It is assumed that the maturation of the digestive tract in Eurasian perch larvae finishes around 21–30 DPH and only from that moment does the intestine reach an ‘adult mode’ of digestion^68^. However, for all the digestive enzymes, Eurasian perch show specific activities as early as just after hatching^41^. In this study, it was observed that the expression of *amy*, *lpl*, *try* and *pga*, as well as the specific activities of amylase, lipase, trypsin, pepsin and LAP were detectable as soon as mouth opening (0–2 DPH) and the specific activities of those digestive enzymes showed similar trends as described in Dąbrowski^13^ and Cuvier Peres and Kestemont^41^. It was reported that in domesticated, adult Eurasian perch, the expression of over 200 genes associated with the digestion process were down-regulated when compared to wild specimens^28^. However, in the present study, the specific activities of digestive enzymes and the expression of their genes in wild and domesticated Eurasian perch larvae suggest that digestion capability is higher in wild fish than in domesticated fish. This suggests that other processes than only the production and activity of digestive enzymes are modified by domestication. This should be considered in future, more detailed research.

The results of the present study indicate that the first selection process following the challenging of the larvae by the type of food occurs in the larval period and most probably affects the rest of the fish’s life. Interestingly, the expression of digestive enzymes seems unlikely to be related to growth potential. In slow- and fast-growing Coho salmon, *Oncorhynchus kisutch*, specimens, there were no changes in specific digestive enzyme activity^69^. Significant changes in growth between Eurasian perch larvae from wild and domesticated spawners were observed only after food replacement. From that moment on, higher growth was observed in domesticated fish, despite lower specific activity of digestive enzymes and lower expression of genes encoding digestive enzymes. This suggests that the production of digestive enzymes does not matter when easily digestible high-quality food, such as *Artemia*, is offered and supports the hypothesis of this study that the type of food is the main challenging factor in intensive culture conditions affecting fish survival.

The environment in which farmed fish live (e.g. RAS) is quite specific and is completely different from natural habitats. All elements of farmed conditions exist only to intensify the production volume and to minimise production costs^70^. The feeding of Eurasian perch larvae in RAS is characterised by high ‘nutritional monotony’. In early stages, larvae are fed mostly with *Artemia* sp. nauplii (with a rather constant composition). After weaning onto commercial feed, it becomes their only source of food until the end of the production process^19^. The digestive tract of domesticated larvae, due to such a small variety of food available, is always prepared to digest non-diverse food and does not need to exhibit such high specific enzymatic activity (and gene expression) as the digestive tracts of wild fish. Since these domesticated fish larvae are no longer under strong selection pressure to maximise the absorption of limited resources, these enzymes and metabolic pathways may be more effective during digestion and they are less active in most cases^28^. On the contrary, there were specific activities of pepsin (and expression of *pga*) and LAP in domesticated Eurasian perch larvae at the end of the experiment. Because domesticated larvae (after weaning) need to digest only high-protein feeds, these enzymes showed a higher specific activity than in wild larvae. In the wild, fish need to be adapted to the digestion of very diverse food, not only high protein food. Wild-living larvae have access to a wide range of zooplankton organisms (with a very different composition), later shifting to benthic organisms and after reaching about 14–18 cm, the adults become exclusively predatory fish^71^. That is why various digestive enzymes are necessary to extract as many nutrients as possible from a limited (and very often not continuous) food supply^28^. Similar dependencies in relation to digestive enzymes have been reported, for example, in a number of passerine birds and some commercial poultry species^72^. All of these findings, along with the results obtained in this study, indicate that domestication processes affect the composition of digestive enzymes, rather than their amount in Eurasian perch.

Stress affects the functioning of the entire organism, as well as the digestive tract, and it could cause, for example, changes in stomach and intestinal secretion and changes in absorption capacity in the intestine^73,74^. Stress can also negatively affect the modification of the intestinal microflora, which - if functioning properly - supports digestion, metabolism and provides significant protection against pathogens^74,75^. It was shown that in Eurasian perch exposed to stress caused by the presence of predators and food shortages that the number of ‘favourable’ intestinal microflora decreased significantly, which affects overall fish welfare^74^. The initial acquisition of domesticated specimens directly from wild progenitors is the unintentional selection of fish which can survive and reproduce in the conditions provided. In this process of unintentional selection, stress resistance is probably one of the most important traits being preserved. That is why several generations of fish grown exclusively in captivity improved the survival rate and stress response^28–30^. Therefore, it should be speculated that because domesticated Eurasian perch larvae were more resistant to stress (caused by artificial rearing conditions) than wild ones, their digestive tracts functioned better. This could be one of the explanations for why domesticated larvae were larger at the end of the experiment and showed a much better survival rate than wild larvae. Also of interest is the fact that even at such a young age, larvae can respond to stress with increased mortality. Nevertheless, this phenomenon requires more detailed research.

## Conclusion

Fish domestication, despite being investigated for decades, still raises many questions, but the present study clearly shows that the effect of the domestication process in Eurasian perch can be observed from the very first days of larvae life. It can be suggested that the differences observed in the present study are stemming from either phenotypic plasticity^75^, epigenetic pattern^76^ or maternally derived modification of the transcriptomic ‘cargo’ determining embryonic and larval performance in finfishes^20^. Besides, the simulataneous involvement of all the three proceses in conditioning the differences between the wild and domesticated stocks can not be excluded. However, regardless the mechanisms underlying the modifications of the digestion capability observed in both populations studied, the results show (for the first time, for freshwater fish larvae) that the larvae from domesticated fish grew faster and exhibited a higher survival rate, despite the lower specific activity of most major digestive enzymes and the lower expression of genes encoding digestive enzymes. However, to fully understand this phenomenon it is necessary to examine the potential involvement of the stress response and immune response in the process of adaptation to the environment of both domesticated and wild Eurasian perch larvae, as they may be involved in the observed differences in growth and survival. Nevertheless, the obtained data allow us to conclude that rearing Eurasian perch larvae obtained from domesticated females is definitely more favourable for the aquaculture sector. This knowledge may be crucial in further studies on selective breeding, as well as commercial selection operations of Eurasian perch, allowing further expansion of commercial production of this species^2^.

## Supplementary information

Suplementary information.

## Data Availability

The obtained perch *pga* mRNA sequence was deposited in GenBank (under accession number MH509190).
