# Author Correction: Domestication process modifies digestion ability in larvae of Eurasian perch (*Perca fluviatilis*), a freshwater Teleostei

**DOI:** 10.1038/s41598-020-62689-2

**Published:** 2020-04-07

**Authors:** Katarzyna Palińska-Żarska, Maciej Woźny, Maciej Kamaszewski, Hubert Szudrowicz, Paweł Brzuzan, Daniel Żarski

**Affiliations:** 1 Department of Ichthyology and Aquaculture, University of Warmia and Mazury, Oczapowskiego 5, 10-719 Olsztyn, Poland; 2 Department of Environmental Biotechnology, University of Warmia and Mazury, Słoneczna 45G, 10-709 Olsztyn, Poland; 3 Departament of Ichthyology and Biotechnology in Aquaculture, Institute of Animal Sciences, University of Life Sciences, Ciszewskiego 8, 02-786 Warsaw, Poland; 4 Department of Gametes and Embryo Biology, Institute of Animal Reproduction and Food Research, Polish Academy of Sciences, Tuwima 10, 10-748 Olsztyn, Poland

Correction to: *Scientific Reports* 10.1038/s41598-020-59145-6, published online 10 February 2020

This Article contains an error in Figure 2 where the average length for Domesticated and Wild Larvae have been incorrectly plotted. The correct Figure 2 appears below as Fig. 1.

**Figure 1:**
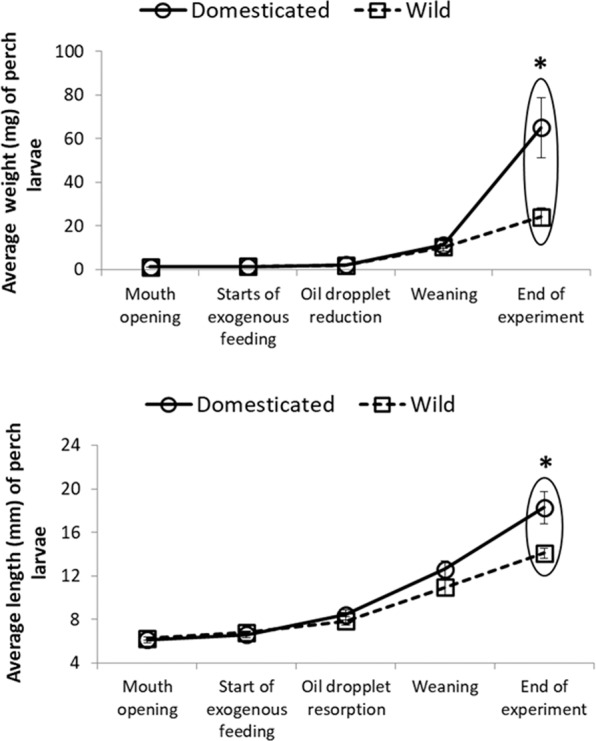
.

