# Supplementary material for: Domestication process modifies digestion ability in larvae of Eurasian perch (Perca fluviatilis), a freshwater Teleostei
Source: Sci Rep. 2020 Feb 10;10:2211. doi: 10.1038/s41598-020-59145-6 (PMC7010758; doi:10.1038/s41598-020-59145-6)

Domestication process modifies digestion ability in larvae of Eurasian perch (*Perca fluviatilis*), a freshwater Teleostei

Palińska-Żarska Katarzyna[1]*, Woźny Maciej[2], Kamaszewski Maciej[3], Szudrowicz Hubert [3], Brzuzan Paweł[2], Żarski Daniel[4]*

[1]*Department of Ichthyology and Aquaculture, University of Warmia and Mazury, Oczapowskiego 5, 10-719, Olsztyn, Poland*

[2]*Department of Environmental Biotechnology, University of Warmia and Mazury, Słoneczna 45G, 10-709, Olsztyn, Poland*

[3]*Departament of Ichthyology and Biotechnology in Aquaculture, Institute of Animal Sciences, University of Life Sciences, Ciszewskiego 8, 02-786, Warsaw, Poland*

[4]*Department of Gametes and Embryo Biology, Institute of Animal Reproduction and Food Research, Polish Academy of Sciences, Tuwima 10, 10-748 Olsztyn, Poland*

* Corresponding authors, contributed equally:

katarzyna.palinska@uwm.edu.pl

d.zarski@pan.olsztyn.pl

Supplement 1. RT-qPCR efficiency and specificity confirmed by dissociation analysis and gel electrophoresis of the primer pairs together with their expression variance.

Name	Adenosine kinase-like [*adk*]	Alpha-amylase [*amy*]
Specificity	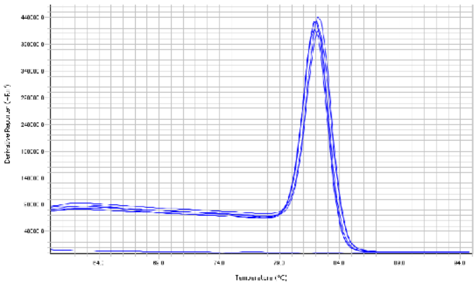	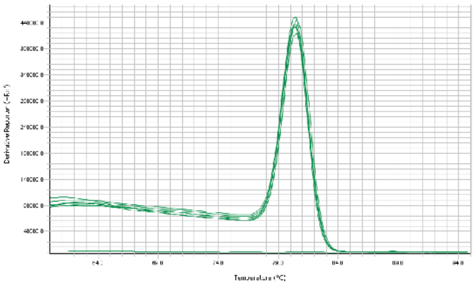
Tm [°C]	82.1	80.4
Standard curve	y=20.295362x–3.614079<br>R^2=0.982122	y=21.464764x–3.485346<br>R^2=0.991598
S.D. [±Cq]	0.72	1.84
M-value	0.67	1.32
Name	Lipoprotein lipase [*lpl*]	Pepsinogen [*pga*]
Specificity	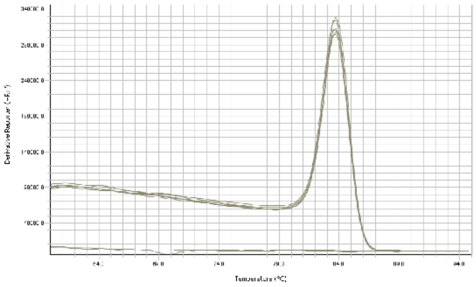	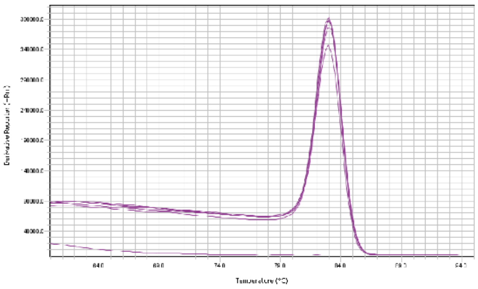
Tm [°C]	83.7	83.0
Standard curve	y=21.023941x–3.234976<br>R^2=0.985577	y=21.031958x–3.644546<br>R^2=0.994482
S.D. [±Cq]	0.89	4.92
M-value	0.67	2.65
Name	Trypsin [*try*]	
Specificity	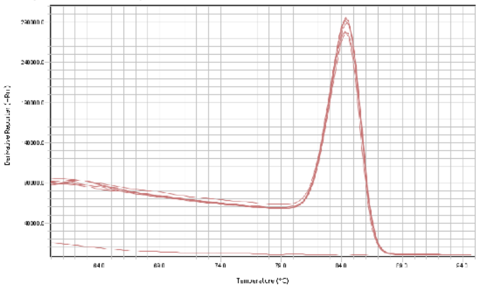	
Tm [°C]	84.5	
Standard curve	y=20.660248x–3.271258<br>R^2=0.993013	
S.D. [±Cq]	2.87	
M-value	1.80	

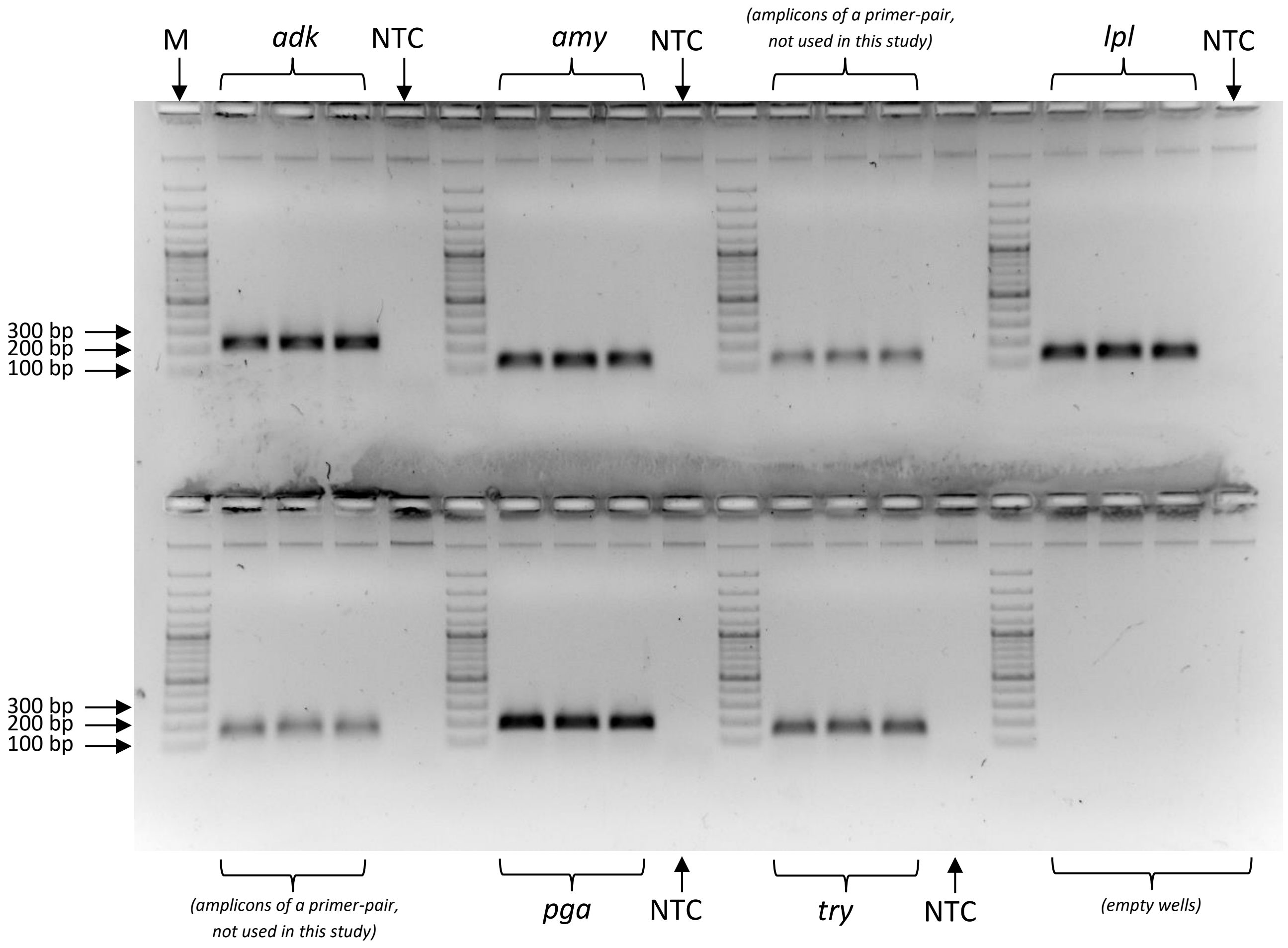

Abbreviations:

M – molecular-weight size marker (100 bp DNA ladder);
NTC – no template control;

Supplement 2. Gene expression of digestive enzymes of Eurasian perch larvae at selected development steps. The graph bars marked with an asterisk showed significant differences ($p < 0.05$). *pga* – pepsinogen, *amy* – amylase, *lpl* – lipase, *try* – trypsin

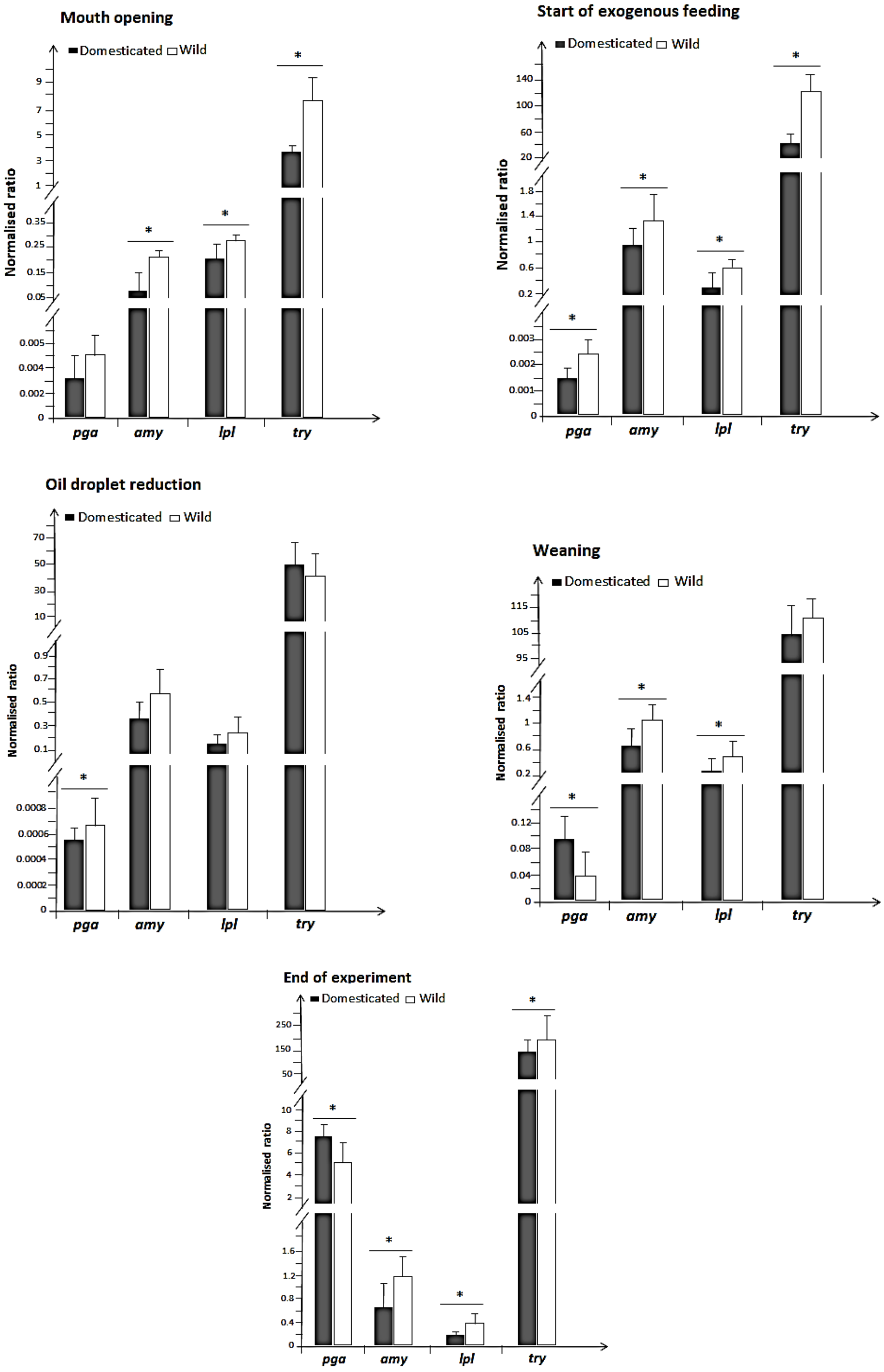

Supplement 3. Specific digestive enzymes activities of Eurasian perch larvae at selected development steps. The graph bars marked with an asterisk showed significant differences ($p < 0.05$).

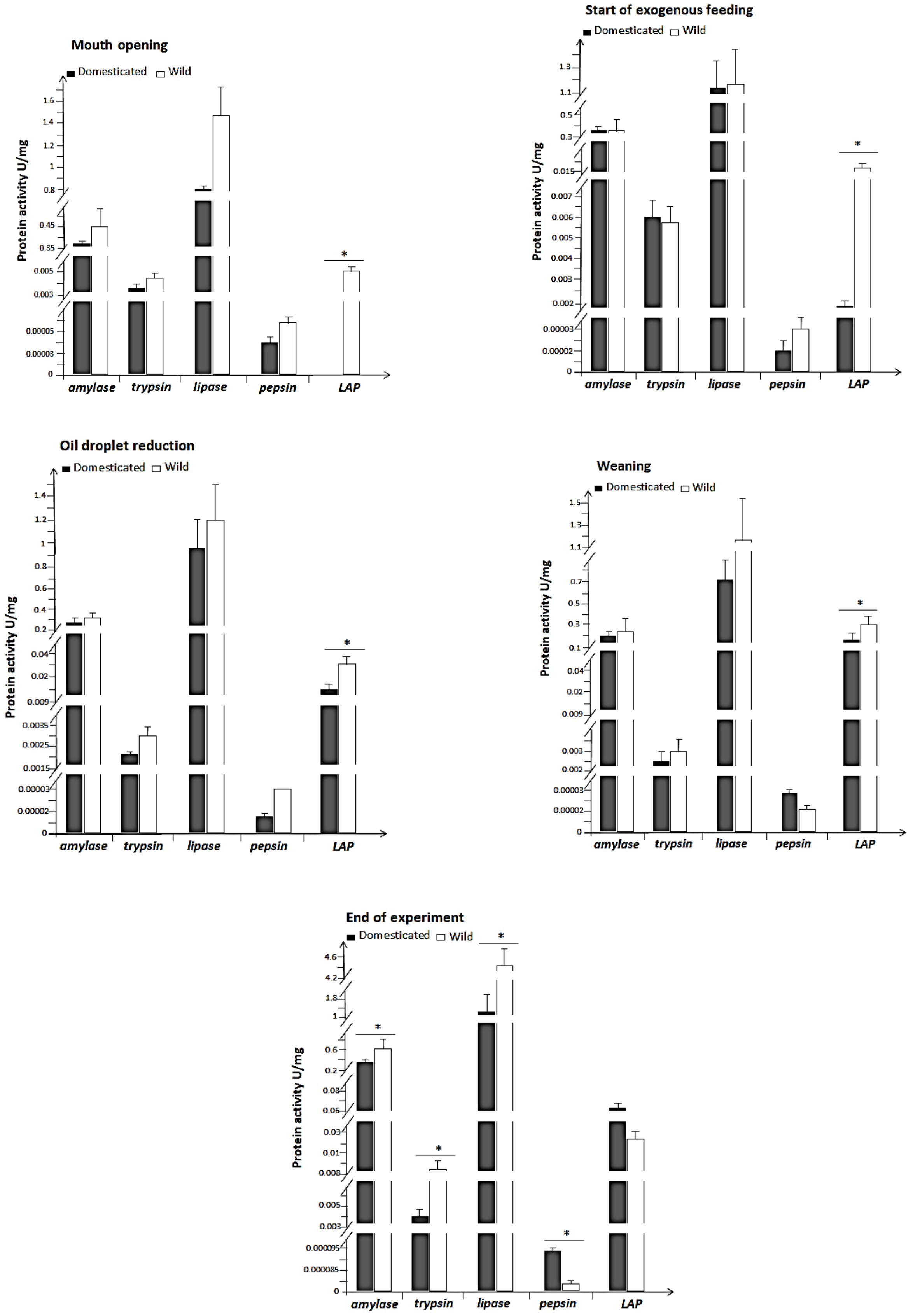

Supplement: Supplementary file 1 — Suplementary information. [file 41598_2020_59145_MOESM1_ESM.pdf]
